# ST534: the new sequence type of Corynebacterium diphtheriae causing diphtheria in Jakarta and surrounding areas, Indonesia

**DOI:** 10.3906/sag-1909-4

**Published:** 2020-02-13

**Authors:** Sunarno SUNARNO, Yuni RUKMINIATI, Ratih Dian SARASWATI

**Affiliations:** 1 Center for Research and Development of Biomedical and Basic Health Technology, National Institute of Health Research and Development, Ministry of Health, Jakarta Indonesia

**Keywords:** *Corynebacterium diphtheriae*, diphtheria, Jakarta, multilocus sequence typing

## Abstract

**Background/aim:**

The aim of this study was to find out characteristics and patterns of the spread of *Corynebacterium diphtheriae* isolated from Jakarta and the surrounding areas, using the whole genome sequencing (WGS) technique and multilocus sequence typing (MLST) approach.

**Materials and methods:**

The study samples consisted of 86 *C. diphtheriae* isolates, which were isolated from diphtheria patients and close contacts of patients. The DNA sequencing was carried out using the WGS technique. Data conversion applied the U-gene software. Molecular typing was conducted through the MLST approach, then followed by online data analysis.

**Results:**

The results showed that as many as 43 (50%) of all samples examined were new types with the same allele profile, namely 9-1-13-4-3-3-4. New sequence type *C. diphtheriae* is registered in the MLST global database as ST534 based on the allele profile. The *tox* gene analysis in 43 isolates with ST534 indicated that there were three mutation positions, all of which were silent mutations.

**Conclusion:**

The main cause of diphtheria in Jakarta and the surrounding areas is a new sequence type of *C. diphtheriae* registered as ST534.

## 1. Introduction

Diphtheria is an acute infectious disease which generally attacks the upper respiratory tract with the typical symptoms of pseudomembranous formation in the focal area of ​​infection followed by a systemic picture due to diphtheria toxin [1,2]. Diphtheria is still a significant health problem in many parts of the world, including Indonesia [3,4]. Data from the Ministry of Health and the World Health Organization (WHO) record that, in the past few years, Indonesia ranks as the second to fourth country in the world with the most cases of diphtheria [5,6]. The spread of the cases extends to almost all provinces. Cases of diphtheria even increased at the end of 2017 until the beginning of 2018. Most cases come from Jakarta and the surrounding areas (Banten and West Java) and East Java [7].

Various efforts were made to overcome diphtheria, including promotive, preventive, and curative efforts. However, new cases continue to be found [8]. There are concerns about the emergence of new variants of bacteria that are more virulent or resistant to vaccines and antibiotics due to mutations, similar to what occurs in other bacteria [9–12]. In this case, it is necessary to carry out the molecular typing of diphtheria-causing bacteria in Indonesia, especially in Jakarta and the surrounding areas. Molecular typing is important to find out the characteristics of bacteria and the spread pattern of disease as well as to evaluate the success of the efforts that have been made [13]. In addition, this activity can be implemented to identify the presence or absence of new circulating variants [14].

Diphtheria is caused by three bacterial species fused in the genus *Corynebacterium* that are *Corynebacterium diphtheria*, *Corynebacterium ulcerans*, and *Corynebacterium pseudotuberculosis* [15]. Molecular typing can be done using several types of methods [16]. In this case, ribotyping is the gold standard for molecular typing of bacteria that cause diphtheria. However, ribotyping has limitations in terms of flexibility. Multilocus sequence typing (MLST) is an alternative with differentiation capabilities equivalent to ribotyping [17]. This study describes the results of molecular typing of a diphtheria-causing bacterium (*C. diphtheriae*) isolated from diphtheria in Jakarta and surrounding areas by the MLST approach.

## 2. Materials and methods 

### 2.1. Time, place, and research sample

The study was conducted at the Bacteriology Laboratory (Prof. Dr. Sri Oemijati Laboratory for Infectious Disease Research), Research Center for Biomedical and Basic Health Technology, in 2018. Samples were in the forms of 86 stored isolates of *C. diphtheriae* that were isolated from diphtheria cases and close contacts of patients in Jakarta and its surrounding areas (Banten and Jabar/West Java) in 2010–2017. The stored isolates were recultured on blood agar and incubated at 37 °C for 24 h. The colonies were harvested and put in a tube containing 0.5 mL of Aquadest for DNA extraction.

The sample proportion per province can be seen in the Figure below.

### 2.2. DNA sequencing

The DNA sequencing was carried out using the next-generation sequencing (NGS) approach. The DNA extraction was conducted with a commercial QIAamp DNA Mini Kit (QIAGEN) following the manufacturer’s procedure. The quality and quantity of DNA were measured before the sample preparation for the DNA sequencing stage using the MiSeq (Illumina) machine. The sample preparation included genomic DNA tagging, amplify libraries, clean up libraries, normalization libraries, and pool libraries. The sequencing process was run by the MiSeq (Illumina) machine by calculating the maximum number of isolates carried out in one process calculated according to the manufacturer’s formula.

### 2.3. Data analysis

Data in the format of “bam” were read, analyzed, and converted into FASTA format using U-gene software. Molecular typing was done with the MLST approach. The profiling of 7 loci was performed and sequence type determination was conducted online via the MLST global database. Further analysis to determine the suitability of bacterial strains with vaccines used in vaccination programs was carried out using BioEdit software. The DNA sequence of the *tox* gene encoding diphtheria toxin synthesis in the sample was compared with the reference strain of *C. diphtheriae* PW8, which was used as the vaccine seed.

## 3. Results

### 3.1. Allele profile and sequence type

A total of 43 (50%) of the 86 isolates examined showed the same allele profile, namely 9-1-13-4-3-3-4. Sequence type (ST) cannot be specified based on the existing database. This shows that the isolate is a new type that has never been reported before. Determination of sequence types is done by registering allele profiles to the MLST global database. The new sequence type was determined and registered with the code ST534. The distribution pattern of isolates with ST534 by region and year of isolation can be seen in Table 1.

**Figure 1:**
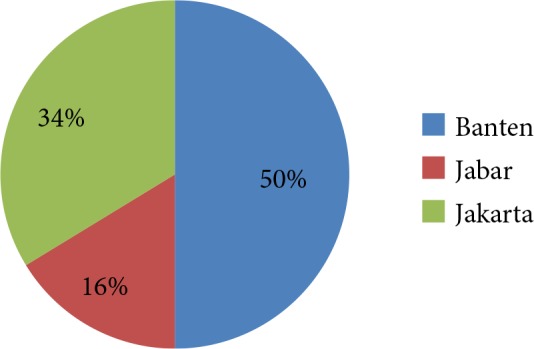
Proportion of samples based on the area of origin of isolates.

**Table 1:** Proportion of ST534 by province and year of isolation.

Year	Banten	Jakarta	West Java	Total
2010	2	-	-	2
2011	3	-	-	3
2012	2	1	-	3
2013	-	-	-	0
2014	3	-	-	3
2015	2	1	-	3
2016	-	3	7	10
2017	9	9	1	19
Total	21 (49%)	14 (32%)	8 (19 %)	43 (100%)

Table 1 shows that bacterial circulation tends to increase from year to year, although it was not found in 2013. The proportion of the spread in each province is proportional to the number of research samples in each province.

### 3.2. Analysis of tox genes

The results of the alignment of the *tox* gene (1683 bp) from 43 isolates with ST534 can be seen in Table 2.

**Table 2:** Variation and location of tox gene mutations in 43 samples with ST534.

Type of bacteria	Mutation location		
	84th base	415th base	705th base
C. diphtheriae PW8 (reference)	T	T	G
Type 1	T	T	G
Type 2	T	C	A
Type 3	C	C	A

Table 2 shows that the mutation pattern of the *tox* gene can be grouped into 3 types and mutations occur at 3 positions from the 1683 nucleotide bases that set the *tox* gene. 

Predictions of changes in amino acids caused by DNA mutations can be seen in Table 3.

**Table 3:** Prediction of codon translation at mutation location.

Mutationlocation	Change in base	Change inamino acid	Type of mutation
84 th Base	GAT→GAC	Asp→Asp	Silent mutation
415 thBase	TTG→CTG	Leu→Leu	Silent mutation
705 thBase	AGG→AGA	Arg→Arg	Silent mutation

Table 3 shows no amino changes due to *tox* gene mutations from all samples.

## 4. Discussion

Diphtheria is an infectious disease through direct contact with patients or carriers and indirect contact with the environment contaminated with the causative bacteria. The spread of disease is closely related to human mobility [18]. This study involved three provinces with very high population mobility, including Jakarta, Banten, and West Java. The most samples came from the Banten region, whereas the samples from West Java were the least (Figure) as they only came from buffer zones (Depok, Bogor, and Bekasi), excluding other regions. Therefore, the number of research samples did not represent the actual number of diphtheria cases in each province.

Molecular typing used in this study was the MLST method. This method has proven reliable for the needs of molecular typing of various types of bacteria, including diphtheria-causing bacteria [17,19]. In diphtheria-causing bacteria, MLST differentiation ability is equivalent to ribotyping, which is the gold standard for molecular typing [17]. The MLST approach to diphtheria-causing bacteria is based on analysis of DNA sequences at 7 loci (7 genes), including *atpA*,* dnaE*,* dnaK*, *fusA*,* leuA*,* odhA*, and *rpoB*. Each allele profile at each locus is sorted to get a sequence type. In this study, ST534 was determined based on the allele profile of the 7 loci. The description of the existence of ST534 almost every year, and even being likely to increase (Table 1), indicates that disease prevention efforts have not been optimal. In addition, 43 ST534 isolates (50% of 86 isolates examined) illustrate that this strain is the main cause of diphtheria in Jakarta and surrounding areas. The proportion of isolates from each province explained that the cases of diphtheria in Jakarta and surrounding areas were related to one another. This strengthens the statement that population mobility greatly affects the spread of diphtheria.

ST534 is a new type sequence, but with allele profiles that already exist in the MLST global database. To find out whether the strain has a higher virulence and immune level, analysis of the *tox* gene encoding diphtheria toxin was performed, as the main virulence factor of bacteria (Tables 2 and 3). The level of immunity referred to here is immunity to the vaccine (antibodies formed by vaccination). This is predicted to occur if the DNA sequence of the *tox* gene from the isolate is different from the DNA sequence of the *tox* gene from the vaccine seed (strain PW8). This is seen in diphtheria cases in developed countries with high vaccination coverage where diphtheria caused by *Corynebacterium ulcerans* tends to increase [20,21], accompanied by the fact that the sequence of the *tox* gene of* C. ulcerans* differs significantly compared to *C. diphtheriae* [22,23].

## Acknowledgment

We would like to thank all of those who played a role in the study and provided input for this manuscript, especially Kambang Sariadji, Nelly Puspandari, Fauzul Muna, Khariri, Bambang Heriyanto, Sarwo Handayani, and Rita Marleta Dewi.
